# Multifaceted anti-infective efficacy of *Alstonia scholaris* (Mizoram chemotype) against fungal, malarial, and intestinal helminth pathogens: *in vitro* validation and molecular docking insights

**DOI:** 10.14202/vetworld.2026.2023-2037

**Published:** 2026-05-16

**Authors:** Lalngaihmanawmi Lalngaihmanawmi, Pawi Bawitlung Lalthanpuii, Bawitlung Lalruatfela, Lal Nundanga, Kholhring Lalchhandama

**Affiliations:** 1 Department of Botany, Government Kolasib College, Kolasib 796081, Mizoram, India; 2 Department of Forestry, Mizoram University, Aizawl 796004, Mizoram, India; 3 Department of Life Sciences (Zoology), Pachhunga University College, Aizawl 796005, Mizoram, India

**Keywords:** anthelmintic, antifungal, antimalarial, drug resistance, medicinal plant, molecular docking, parasite, triterpenoids

## Abstract

**Background and Aim:**

The increasing prevalence of drug-resistant fungal, malarial, and helminth infections necessitates the identification of novel, broad-spectrum therapeutic agents. *Alstonia scholaris*, widely used in Mizo traditional medicine, has been reported for treating microbial and parasitic infections. This study aimed to evaluate the multifaceted anti-infective efficacy of a geographically distinct Mizoram chemotype of *A. scholaris* through *in vitro* assays and molecular docking analyses.

**Materials and Methods:**

Methanolic bark extract of *A. scholaris* was assessed for antifungal activity against *Aspergillus fumigatus*, *Candida albicans*, and *Neocosmospora keratoplastica* using the agar poison technique. Antimalarial activity was tested against chloroquine-sensitive (3D7) and multidrug-resistant (K1) strains of *Plasmodium falciparum* using a SYBR Green I-based fluorescence assay. Cytotoxicity was evaluated in Vero C1008 cells using the 3-(4,5-dimethylthiazol-2-yl)-2,5-diphenyltetrazolium bromide (MTT) assay. Anthelmintic efficacy was determined against *Raillietina echinobothrida* using histological and scanning electron microscopy analyses. Bioactive compounds were identified by gas chromatography-mass spectrometry (GC-MS), followed by molecular docking against key pathogen targets.

**Results:**

The extract demonstrated significant antifungal activity, with *N. keratoplastica* being the most susceptible species. Antimalarial activity showed comparable efficacy against both *P. falciparum* strains, with IC_50_ values of 43.40 µg/mL (3D7) and 45.60 µg/mL (K1), and a low resistance index of 1.05. Cytotoxicity analysis indicated high CC_50_ (>100 µg/mL), suggesting safety, although selectivity indices were moderate. Anthelmintic activity revealed concentration-dependent effects, comparable to albendazole (p > 0.05). Histological and ultrastructural analyses confirmed severe tegumental and internal tissue damage in parasites. GC-MS identified triterpenoids, predominantly α-amyrin and kolavenol. Docking studies revealed strong binding affinities of α-amyrin with fungal sterol 14α-demethylase (CYP51, at –10.4 kcal/mol), malarial S-adenosyl-L-homocysteine hydrolase (SAHH, at –10.0 kcal/mol), and helminth glutamate-gated chloride channel (GluCl, at −9.4 kcal/mol), supporting multi-target activity.

**Conclusion:**

The Mizoram chemotype of *A. scholaris* exhibits broad-spectrum anti-infective potential with antifungal, antimalarial, and anthelmintic activities. The predominance of triterpenoids, particularly α-amyrin and kolavenol, distinguishes it from other chemotypes and underpins its pharmacological profile. These findings validate its traditional use and highlight its potential as a source of novel anti-infective agents, warranting further *in vivo* and mechanistic studies.

## INTRODUCTION

The global burden of diseases due to microbial and parasitic infections remains a persistent medical problem and a continuing challenge in clinical and veterinary practice despite advancements in disease management and the development of numerous medications. In fungal diseases, the use of multiple medications for different infections has contributed to the emergence of life-threatening forms of pathogens that were previously considered manageable [1]. Clinical use of many drugs for mild to severe ailments is now recognized to compromise the immune system, thereby facilitating increased virulence of invasive fungi [2]. A global estimate indicates that fungal pathogens are increasing in both prevalence and infection severity [3].

A more serious concern arises from parasitic infections, particularly malaria and helminthiases. *Plasmodium falciparum*, an apicomplexan protozoan responsible for malignant malaria in humans, remains the deadliest single parasite species [4], accounting for more than 280 million infections and over half a million deaths annually [5]. Helminthiasis refers to parasitic infections caused by diverse helminths, broadly categorized into nematodes (roundworms), cestodes (tapeworms), and trematodes (flukes). Collectively, these infections represent the most prevalent infectious diseases, affecting billions of people and animals of veterinary importance [6, 7]. The consequences extend beyond human and animal health, leading to significant socioeconomic burdens, including increased morbidity and reduced agricultural productivity [8, 9]. The most critical barrier to effective infection management is the emergence and spread of drug resistance across all pathogen types [10, 11]. Concurrently, the stagnation in the development of new pharmaceutical agents underscores the urgent need for effective and safe therapeutic alternatives.

Some of the most significant advances in medicine have been derived directly or indirectly from medicinal plants. Historically, quinine, obtained from *Cinchona pubescens*, represents one of the earliest and most life-saving drugs. Its semi-synthetic and synthetic derivatives, collectively known as quinolines, became first-line treatments for falciparum malaria and other malaria forms, saving millions of lives [12]. This marked the first discovery of a compound capable of curing an infectious disease [13], generating optimism that malaria could be controlled [14]. However, this optimism was undermined by the emergence of antimalarial resistance in the late 20th century [15], leading to the resurgence of malaria as a major health concern [16].

The discovery of artemisinin from *Artemisia annua* revitalized malaria control strategies. Artemisinin and its derivatives are currently the primary antimalarial agents [17]. However, resistance in *P. falciparum* has again emerged, posing a significant challenge [18]. Notably, quinoline and artemisinin derivatives exhibit additional pharmacological properties, including anthelmintic and antimicrobial activities [19, 20]. Therefore, the discovery of new drugs continues to rely heavily on traditionally used antimalarial and anthelmintic plants [21].

Mizoram, located in Northeast India, is part of the Indo-Burma biodiversity hotspot and possesses rich biodiversity in flora and fauna [22]. One medicinal plant widely used in the Mizo traditional system is *Alstonia scholaris* (synonym *Echites scholaris*) (family Apocynaceae). Extracts from its bark and leaves are traditionally used to treat asthma, cardiac disorders, diarrhea, dysentery, hypertension, intestinal worm infections, malaria, snake bites, and typhoid fever [23, 24]. If these traditional uses are validated scientifically, the plant could represent a valuable source of bioactive compounds with broad-spectrum therapeutic potential.

An important distinguishing feature of the Mizoram variety of *A. scholaris* is its unique morphology, characterized by a circular arrangement of eight leaves per node, a trait not reported in other regions. In contrast, varieties from mainland India typically have seven leaves per node, whereas other Asian variants may have four, five, or ten leaves [25]. Such morphological differences may reflect underlying biochemical variations that influence pharmacological properties. For example, artemisinin is abundant in *A. annua* from China but not consistently present in the same species from other regions [26]. This highlights the importance of evaluating geographically distinct chemotypes.

This study is novel in providing a comprehensive evaluation of the Mizoram chemotype of *A. scholaris* against multiple pathogens, including fungi, drug-resistant malarial parasites, and parasitic cestodes. The investigation integrates *in vitro* assays with molecular docking analyses to assess bioactivity and explore potential mechanisms of action.

Despite extensive studies on *A. scholaris* from various geographical regions, there is a lack of systematic investigation on the Mizoram chemotype, particularly regarding its multi-target anti-infective potential. Previous studies have primarily focused on alkaloid-rich chemotypes and often evaluated single-pathogen activity, leaving a critical gap in understanding cross-pathogen efficacy. Additionally, there is limited information on the role of non-alkaloid compounds, such as triterpenoids, in mediating broad-spectrum pharmacological effects. Furthermore, integrative approaches combining *in vitro* validation with computational molecular docking for this specific chemotype remain unexplored.

The present study aims to evaluate the multifaceted anti-infective efficacy of the Mizoram chemotype of *A. scholaris* by assessing its antifungal, antimalarial, and anthelmintic activities using *in vitro* assays. In addition, the study seeks to identify the major bioactive compounds using gas chromatography-mass spectrometry (GC-MS) and to investigate their molecular interactions with key pathogen targets through docking analyses. This integrated approach is intended to validate traditional medicinal claims and to identify potential lead compounds for the development of novel anti-infective therapeutics.

## MATERIALS AND METHODS

### Ethical approval

The use of animal-derived parasitic material was approved by the Institutional Animal Ethics Committee of Pachhunga University College, Aizawl, Mizoram, India (approval number PUC-IAEC-2022-Z02, dated 15/03/2022). The study involved the collection of adult *Raillietina echinobothrida* from the intestines of slaughtered chickens and did not involve experimental infection, live animal experimentation, or any invasive procedure on live animals.

All procedures for parasite collection, handling, maintenance, treatment, fixation, histological processing, and scanning electron microscopy were conducted in accordance with institutional ethical guidelines and standard laboratory biosafety practices. The use of parasite material was limited strictly to the objectives of evaluating the anthelmintic efficacy of *A. scholaris* bark extract. No human participants or clinical human samples were used in this study.

### Study period and location

The study was conducted from November 2022 to May 2025. Plant specimens of *A. scholaris* were collected from Lungdai, Mizoram, India (latitude 23°52′ N, longitude 92°44′ E, altitude 1200 m above sea level). Antifungal, anthelmintic, histological, scanning electron microscopy, chemical profiling, and molecular docking analyses were performed at Pachhunga University College, Aizawl, Mizoram, India. Antimalarial and cytotoxicity assays were performed at the Central Drug Research Institute (CDRI), Lucknow, Uttar Pradesh, India.

### Study design

This experimental laboratory-based study evaluated the anti-infective potential of *A. scholaris* bark extract against fungal, malarial, and helminth pathogens. The study included the extraction of plant material, antifungal assays, antimalarial assays against *P. falciparum*, cytotoxicity testing using Vero C1008 cells, anthelmintic evaluation against *R. echinobothrida*, histological and scanning electron microscopy observations, GC-MS-based chemical profiling, and molecular docking analysis.

### Plant specimen and extraction

Mature plant parts, including flowers of *A. scholaris*, were collected from a forest in Lungdai, Mizoram, India. Authentication of the specimens (voucher number PUC-A-23-01 dated 25/11/2022) was performed at the Eastern Regional Center of the Botanical Survey of India, Meghalaya, India (BSI/ERC/Tech/2023-24/102 dated 17/05/2023). The bark was cut into small pieces, washed in distilled water, and dried in shade under ambient conditions (25 ± 2°C).

The dried samples were extracted by maceration and Soxhlet extraction using chloroform and methanol. The solvents were evaporated and recycled under vacuum pressure using Buchi Rotavapor® R-100 (Büchi Labortechnik AG, Flawil, Switzerland). The extract yield was calculated using the formula: [W1 ÷ W2] × 100, where W1 is the weight of the final extract and W2 is the weight of the dried sample. The extract was refrigerated at 4°C until use.

### Antifungal assay

The antifungal activity of *A. scholaris* extract was evaluated using the poison plate technique of Grover and Moore [27], with a modified potato-dextrose agar culture method [28]. Subcultures of *Aspergillus fumigatus* (ATCC 204305), *Candida albicans* (ATCC 26790), and *Neocosmospora keratoplastica* (ATCC 36031) were obtained from ATCC, Manassas, Virginia, USA. The fungal cultures were streaked evenly on sterilized agar media in Petri dishes. They were grown for seven days at 27 ± 2°C in a disinfected microbiological chamber (Igene IG-95I, Igene Labserve Pvt. Ltd., New Delhi, India).

Serial dilutions of the *A. scholaris* extract (10, 5, 2.5, and 1.25 mg/mL) were prepared in 20 mL potato-dextrose agar. The agar was kept molten at approximately 50°C. Fungi grown only in the growth medium were maintained as the control. Using a sterile cork borer, 6 mm disks of fully grown fungi were inoculated into the center of the culture plates containing extract-treated and control media. After solidification, the plates were hermetically sealed with parafilm and incubated for seven days at 27 ± 2°C. The growth zones formed radially around the inoculum were measured at two opposite circumferences every 24 h. The inhibitory activity was estimated using the standard formula.

Growth inhibition (%) = [(Growth of control − Growth of treated) ÷ Growth of control] × 100

### Maintenance of malarial parasites

Two isolates of *P. falciparum*, chloroquine-sensitive 3D7 and multidrug-resistant K1, were obtained from the Biodefense and Emerging Infections Research Resources Repository of the National Institute of Allergy and Infectious Diseases, U.S. National Institutes of Health, and maintained at CDRI, Lucknow, India. They were cultivated in complete RPMI 1640 medium containing 8% parasitaemia at the erythrocytic trophozoite stage and 2% erythrocyte volume [29]. The culture medium was supplemented with HEPES, 0.5% AlbuMAX™ II lipid-rich bovine serum albumin (Gibco, Thermo Fisher Scientific, Waltham, Massachusetts, USA), 0.2% D-glucose, 0.2% sodium bicarbonate, 45 mg/L hypoxanthine, 0.25 mg/L fungizone, 50 mg/L gentamycin, and 15 μM hypoxanthine. The cultures were maintained at 37 ± 1°C in a humidified 5% CO_2_ incubator (Forma™ Series II, Thermo Fisher Scientific, Waltham, Massachusetts, USA).

### Antimalarial assay

Antimalarial susceptibility was tested using malaria SYBR Green I-based fluorescence (MSF) [30]. Parasitized erythrocytes at 0.8% parasitaemia and 1% hematocrit in RPMI were exposed to *A. scholaris* extract prepared in serial dilution from 50 μg/mL stock solution. They were cultured in 96-well plates at 37 ± 1°C in a humidified 5% CO_2_ incubator.

Uninfected erythrocytes were maintained as the negative control, and infected cells without drug treatment were maintained as the positive control. One group of parasitized erythrocytes was treated with chloroquine, a reference drug, at 0.05–0.3 ng/μL. After 72 h of culture, 100 μL of lytic buffer containing 1× SYBR Green was added to all samples, and the samples were incubated again for 2 h. Fluorescence intensity was detected at 485 ± 20 nm excitation and 530 ± 20 nm emission using a fluorescence reader (BioTek Synergy™, Agilent Technologies, Inc., Santa Clara, California, USA). IC_50_ was calculated using programmed statistical analysis [31].

### Cytotoxicity test

Cytotoxicity of *A. scholaris* extract was assessed using the MTT reduction assay [31]. Vero C1008 cells were acquired from ATCC (CRL-1586™) and maintained at CDRI. They were seeded in a 96-well plate at 10^4^ cells per well and maintained under the same conditions as the malarial parasites. The cells were treated with different dilutions of the extract. Positive control cells were exposed to podophyllotoxin.

After 72 h of incubation, the samples were mixed with 25 µL of MTT at a stock concentration of 5 mg/mL. After 2 h, absorbance was recorded at 570 nm, from which CC_50_ was calculated. SI was calculated as the ratio of CC_50_ to IC_50_.

### Helminth survival assay

Anthelmintic activity was tested on the intestinal cestode *R. echinobothrida* Mégnin, 1880, collected from chicken intestines [32]. The parasites were maintained in 0.9% phosphate buffered saline (PBS) supplemented with 1% dimethyl sulfoxide (DMS) at 37 ± 1°C. Parasites maintained only in PBS + DMS served as the negative control.

Experimental groups were treated with different concentrations of *A. scholaris* extract: 5, 10, and 20 mg/mL. Positive control groups consisted of parasites treated with albendazole at similar concentrations. After each experiment, the parasites were washed in PBS and fixed in Bouin solution. Dehydration was performed using 30–100% ethanol, then cleared in xylene. Paraffinized blocks were cut at a thickness of 4–5 µm using an auto-microtome (MRM-ST, Medimeas Instruments, Ambala, Haryana, India). The sections were dehydrated again, stained with eosin and hematoxylin, fixed on glass slides, and observed under a Nikon Eclipse image analyzer (Nikon Corporation, Tokyo, Japan).

For scanning electron microscopy, the parasites were fixed in 10% neutral-buffered formaldehyde at 4°C for 4 h. They were dehydrated in increasing grades of acetone. The specimens were immersed in tetramethylsilane for 10 min and evaporated to dryness at 25°C in an air-drying chamber. After coating with gold using an MC1000 ion sputter coater (Hitachi, Ltd., Tokyo, Japan), the electron micrographs were taken with a TM4000Plus II scanning electron microscope (Hitachi, Ltd.).

### Chemical profiling

Identification of bioactive compounds in *A. scholaris* extract was performed using GC-MS with a TRACE™ 1300 ISQ™ LT system (Thermo Fisher Scientific, Waltham, Massachusetts, USA). One microliter of extract dissolved in methanol was injected with helium as the carrier gas. A non-polar TR-5MS column (30 m × 0.25 mm × 0.25 μm) was used. The injection port temperature was set at 280°C, the transfer line and ion source temperatures at 220°C, and the oven temperature was increased from 70°C to 250°C. Thermo Scientific™ Xcalibur™ software (Thermo Fisher Scientific) was used to generate the data. The data were compared with the National Institute of Standards and Technology (NIST) database.

### Computational ligand–receptor modeling

The compounds identified in *A. scholaris* extract were docked against vital receptors in pathogenic fungi, *P. falciparum*, and helminth parasites. The three-dimensional structures of the compounds were retrieved from PubChem, NCBI. The chemical conformations were optimized and the cumulative energies minimized using ChemBio3D Ultra 12.0 (CambridgeSoft Corporation, Cambridge, Massachusetts, USA) with the MMFF94 force field.

The molecular target proteins were acquired from RCSB-PDB, including *Candida albicans* sterol 14α-demethylase (CYP51), *C. albicans* farnesyltransferase, *C. albicans* geranylgeranyltransferase type 1 (GGTase-I), *Plasmodium falciparum* erythrocyte membrane protein 1 (VAR2CSA), and S-adenosyl-L-homocysteine hydrolase (SAHH), *Caenorhabditis elegans* β-tubulin, and *C. elegans* glutamate-gated chloride channel (GluCl). Ligand binding was simulated using AutoDock Vina v1.2.4 (Molecular Graphics Lab, The Scripps Research Institute, La Jolla, California, USA) [33]. MGLTools 1.5.6 (Molecular Graphics Lab) was used to add Kollman charges and polar hydrogens to the proteins. The final configurations were saved in PDBQT files. BIOVIA Discovery Studio Visualizer 2016 (Dassault Systèmes, Vélizy-Villacoublay, France) was used to analyze the final structures.

### Statistical analysis

Comparison of mean differences between treatments and controls was performed using Student’s t-test. Group comparisons were conducted using analysis of variance followed by Tukey’s honest significant difference test. The level of significance was set at p < 0.05. Statistical analysis and graphs were generated using GraphPad Prism version 10.4.1 (Dotmatics, Boston, Massachusetts, USA).

## RESULTS

### Plant extract

The extract yields of *A. scholaris* bark were 5.15% by maceration, 6.36% with chloroform, and 18.35% with methanol. The best-yielding methanol extract was found to contain the greatest variety of compounds and was used for all experiments.

### Antifungal activity

**Figure 1:**
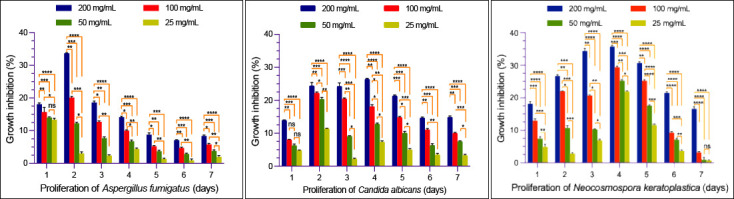
Statistical comparison of the antifungal activity of *Alstonia scholaris* bark extract against *A. fumigatus*, *C. albicans*, and *N. keratoplastica*. Columns represent values in means ± standard error of means (n = 3), **** p < 0.0001, *** p < 0.002, ** p < 0.001, * p < 0.05, and ns = not significant, i.e., p > 0.05.

The antifungal activity of *A. scholaris* bark extract was assessed based on the growth patterns of *A. fumigatus*, *C. albicans*, and *N. keratoplastica*, as tabulated in Table 1. There was a steady proliferation of the three fungi in the culture media for 7 days, and the proliferation rate was determined from the growth zones formed around the sample wells. The growth rate of the control sample, maintained without the plant extract, was highest, as indicated by the increasing number of growth zones each day across all species. The relative growth rate was in the order *A. fumigatus* > *N. keratoplastica* > *C. albicans*. In the plant extract-treated groups, there was no significant inhibition of growth (p ≥ 0.05) on the first day in all species compared to the growth rate of control, and a significant difference appeared after the first day. The percentage of inhibition was calculated for each species from the growth zones of the respective control. Statistical comparison between treatment groups, as shown in Figure 1, indicates that *N. keratoplastica* was evidently the most susceptible species against the plant extract, followed by *C. albicans*. The inhibitory effect on *A. fumigatus* was minimal after the second day.

**Table 1:** Fungal proliferation under control conditions and treatment with *Alstonia scholaris* bark extract. Values are in means ± standard deviations.

Species	Duration	Growth zone (mm)				

		0 (control)	1.25 mg/mL	2.5 mg/mL	5 mg/mL	10 mg/mL
*A. fumigatus*	Day 1	07.21 ± 1.24	06.24 ± 0.78	06.19 ± 0.29	07.69 ± 2.40	05.90 ± 0.67
	Day 2	19.26 ± 0.84	18.67 ± 0.89	16.19 ± 0.69	15.38 ± 0.54	12.62 ± 0.43
	Day 3	29.67 ± 0.45	28.58 ± 0.49	27.01 ± 0.69	25.55 ± 0.54	23.83 ± 0.77
	Day 4	46.23 ± 0.97	44.18 ± 0.44	43.13 ± 0.78	41.60 ± 0.40	39.69 ± 0.47
	Day 5	60.01 ± 0.52	59.21 ± 0.39	57.83 ± 0.61	56.86 ± 0.42	54.79 ± 0.42
	Day 6	72.52 ± 0.53	70.20 ± 0.45	71.08 ± 0.53	69.75 ± 0.46	67.99 ± 0.18
	Day 7	79.98 ± 0.65	78.81 ± 0.75	77.30 ± 0.58	75.72 ± 0.55	73.65 ± 0.72
*C. albicans*	Day 1	06.36 ± 0.46	06.79 ± 1.16	06.46 ± 0.68	06.24 ± 0.07	05.84 ± 0.18
	Day 2	12.57 ± 0.45	11.13 ± 0.20	09.77 ± 0.80	09.49 ± 0.45	10.01 ± 0.65
	Day 3	16.56 ± 0.42	16.19 ± 0.34	15.05 ± 0.26	12.53 ± 0.41	13.17 ± 1.71
	Day 4	26.69 ± 0.45	25.09 ± 0.48	23.53 ± 0.43	21.85 ± 0.81	19.39 ± 0.43
	Day 5	31.27 ± 0.57	29.69 ± 0.43	28.11 ± 0.79	26.63 ± 0.46	24.58 ± 0.39
	Day 6	32.18 ± 0.51	31.04 ± 0.61	30.12 ± 0.81	28.60 ± 0.50	27.45 ± 0.38
	Day 7	33.71 ± 0.54	32.59 ± 0.24	31.17 ± 0.25	30.33 ± 0.41	28.67 ± 0.50
*N. keratoplastica*	Day 1	05.09 ± 1.37	04.71 ± 1.42	04.81 ± 1.01	04.43 ± 0.94	04.17 ± 1.97
	Day 2	15.21 ± 1.23	15.55 ± 0.53	15.50 ± 1.32	12.48 ± 0.25	11.74 ± 0.73
	Day 3	28.11 ± 0.40	26.17 ± 0.56	25.21 ± 0.19	22.30 ± 0.37	18.45 ± 1.57
	Day 4	46.35 ± 1.39	36.21 ± 0.38	34.67 ± 0.57	32.73 ± 0.54	28.81 ± 0.58
	Day 5	57.97 ± 0.70	46.83 ± 0.55	43.68 ± 0.46	39.61 ± 0.51	36.74 ± 0.68
	Day 6	70.84 ± 0.61	68.29 ± 0.76	65.81 ± 0.75	64.29 ± 0.76	55.64 ± 0.57
	Day 7	75.25 ± 2.00	71.74 ± 0.51	71.55 ± 1.24	69.95 ± 0.53	60.28 ± 1.12

### Antimalarial and cytotoxic activity

*A. scholaris* bark extract was almost equally effective against both the drug-sensitive and drug-resistant isolates of *P. falciparum* as shown in Table 2. The IC_50_ values were 43.40 µg/mL against 3D7 and 45.60 µg/mL against K1, both within the antimalarial efficacy category of good or moderate activity. It was remarkable that the plant extract was effective against the multidrug-resistant isolate, while the reference drug, chloroquine, had no effect. The antimalarial resistance index (Ri), calculated as the ratio of the relative effectiveness of drug-sensitive to drug-resistant isolates, was 1.05. This indicates that the plant extract has an extremely low level of potential resistance in the parasites. In comparison, chloroquine shows an extreme level of resistance in K1, with an Ri of 39.85, indicating that it has no pharmaceutical value against this isolate.

**Table 2:** Antimalarial susceptibility and cytotoxicity of *Alstonia scholaris* bark extract and standard drugs.

Sl. no.	Treatment	IC_50_ (µg/mL) 3D7	IC_50_ (µg/mL) K1	Resistance index (Ri)	CC_50_ (µg/mL)
1	*A. scholaris* extract	43.40	45.60	1.05	>100
3	Chloroquine	2.05	81.57	39.85	NA
4	Podophyllotoxin	NA	NA	NA	1.45

Chloroquine = Positive control for antimalarial test, NA = Not applicable, 3D7 = Chloroquine-sensitive strain, K1 = Multidrug-resistant strain, podophyllotoxin = Positive control for cytotoxicity test, Vero = C1008 strain of epithelial cell line from the kidney of an African green monkey.

The cytotoxicity of *A. scholaris* bark extract against Vero C1008 cells is also shown in Table 2. CC_50_ values above 100 µg/mL indicate that the plant extract is non-toxic and safe for normal cells. In contrast, the reference toxin, podophyllotoxin, was extremely toxic with a CC_50_ of 1.45 µg/mL. However, the selectivity indices of the plant extract were low: 6.12 for 3D7 and 5.81 for K1.

### Anthelmintic activity

**Figure 2:**
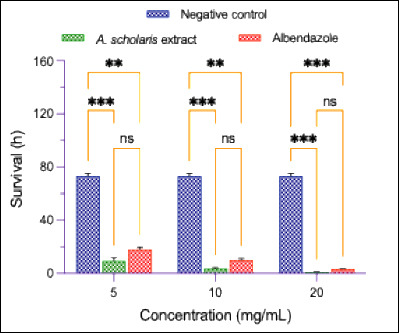
Statistical comparison of the anthelmintic activity of *Alstonia scholaris* bark extract and albendazole against the cestode, *Raillietina echinobothrida*. Columns represent values in means ± standard error of means (n = 3), **** p < 0.0001, *** p < 0.002, ** p < 0.001, * p < 0.05, and ns = not significant, i.e., p > 0.05

The anthelmintic efficacy of albendazole and *A. scholaris* bark extract is summarized in Table 3. Both treatments resulted in concentration-dependent responses in the cestode. The parasites were significantly more sensitive to the drug, albendazole, as indicated in Figure 2. *A. scholaris* bark extract, albeit with less potency, showed significant efficacy even at the lowest concentration tested. However, albendazole and the plant extract were equally effective, i.e., no significant differences (p > 0.05) across all concentrations, with p values of 0.22 for 5 mg/mL, 0.14 for 10 mg/mL, and 0.07 for 20 mg/mL.

**Table 3:** Comparative efficacy of *Alstonia scholaris* bark extract and albendazole against the cestode, *Raillietina echinobothrida*.

Treatment media	Dose (mg/mL)	Survival value (h)	t Stat	t Critical
*A. scholaris* extract	5	18.02 ± 3.18*	20.06	2.78
	10	10.02 ± 2.16*	26.35	3.18
	20	3.42 ± 0.37*	33.88	4.30
Albendazole	5	9.40 ± 3.89*	20.98	2.78
	10	3.74 ± 1.09*	32.41	2.78
	20	1.19 ± 0.43*	34.89	2.78

* Significantly different at p < 0.05 against negative control (n = 3). Values are in means ± standard deviations.

### Histological observations

**Figure 3:**
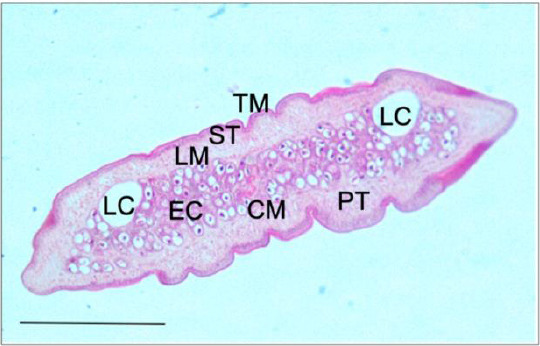
Light micrographs of the histological section of *Raillietina echinobothrida* treated with *Alstonia scholaris* bark extract. The external body surface is the tegument (TM). Beneath it lies the sub-tegument (ST), which remains normal. The longitudinal muscle (LM) and the surrounding parenchyma are diffused. Two prominent vacuoles on the sides are lateral canals (LC). The egg capsules (EC) surrounded by circular muscle (CM) are at the center of the body. (×400, scale bar = 20 μm).

Histological section of the cestode treated with the plant extract indicated several tissue damages as shown in Figure 3. Numerous corrugations on the body surface (the tegument) indicate severe shrinkage that has formed creases and folds. Internally, the most affected anatomical parts are the parenchymatous tissue that hold the muscle and subtegumental layers. The tissues are marked with irregular dots and stains indicating the disintegration of the proteinaceous filaments. Several eggs were empty, indicating loss of cytoplasmic materials, including the nuclei. There are also some parts of the circular muscle that have disappeared, indicating the disintegration of the muscle fibers.

### Scanning electron microscopy

**Figure 4:**
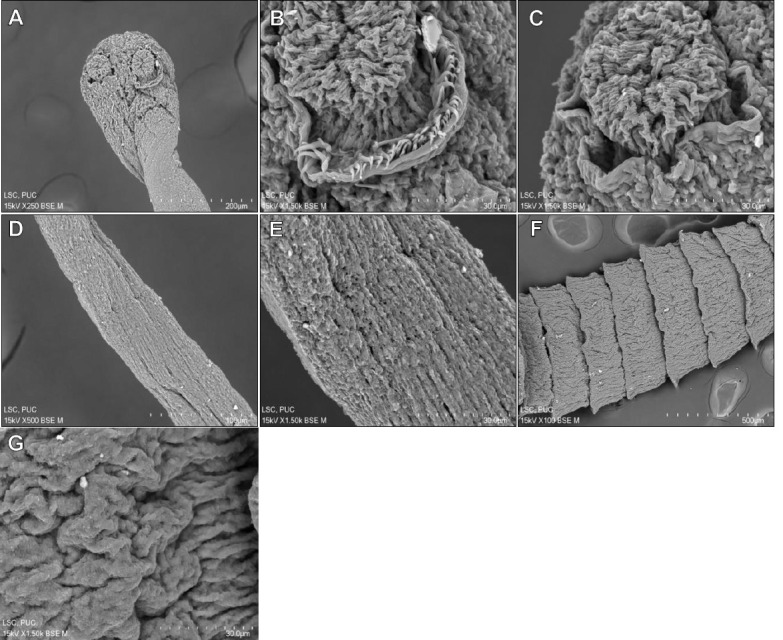
Electron micrographs of the body parts of *Raillietina echinobothrida* treated with *Alstonia scholaris* bark extract. (A) The scolex and neck. (B) The right sucker. (C) The left sucker. (D) Immature body segments toward the anterior region. (E) Magnified details of the tegument of immature body segments. (F) Mature body segments toward the posterior end. (G) Details of mature body segments.

Signature anthelmintic effects of the plant extract were evident under scanning electron microscopy. The anterior bulbous end, the scolex, is full of wrinkles due to shrinkage of the body surface tegument (Figure 4A). Even the two eye-like suckers are completely wrinkled. A magnification of one sucker shows a circular rim having rows of spines (whitish, pointed, and curved structures) at some portion, but some of which are detached at the bottom and totally vanished on the left side (Figure 4B). However, all the spines on one sucker are completely lost (Figure 4C). The tegument in the neck region is totally eroded (Figure 4D), and the smooth surface is entirely replaced by tissue lumps and creases (Figure 4E). Tegumental shrinkage extends throughout the mature body segments, proglottids. Massive folds are visible on all the proglottids (Figure 4F). A closer view of the tegumental surface of a mature proglottid reveals contorted folds due to constriction and the complete loss of surface filaments and microtriches (Figure 4G).

### Compound analysis

**Figure 5:**
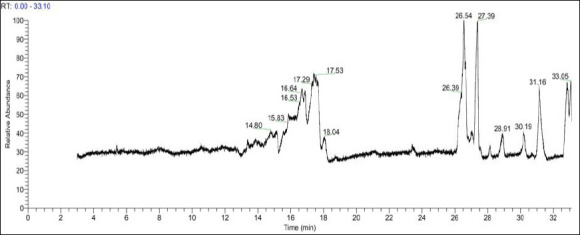
Gas chromatogram of the methanol extract of *Alstonia scholaris* bark.

The gas chromatogram of the extract for identification of bioactive compounds is shown in Figure 5. The peak intensity and mass were used to compare compounds in the NIST library (at similarity score > 90%), which showed that the plant extract is rich in triterpenoids (Table 4). The three most abundant compounds, α-amyrin, kolavenol, and lup-20(29)-en-3-yl acetate, were all triterpenoids. Kolavenol at retention time (RT) 26.54 and α-amyrin at RT 27.39 were identified as the two main bioactive compounds. Kolavenol was additionally detected at RT 26.39, and α-amyrin at RT 31.16. Although at a lower abundance value, lup-20(29)-en-3-yl alcohol was detected at seven RT.

**Table 4:** Compounds identified in *Alstonia scholaris* extract from the National Institute of Standards and Technology (NIST) chemical database using gas chromatography-mass spectrometry (GC-MS).

Peak no.	Retention time (min)	Relative abundance (%)	Compound	Formula	Molecular weight
1	14.80	41.4	Lup-20(29)-en-3-yl acetate	C32H52O2	468
2	15.83	45.5	Lup-20(29)-en-3-yl acetate	C32H52O2	468
3	16.53	56.2	Lup-20(29)-en-3-yl acetate	C32H52O2	468
4	16.64	61.3	Lup-20(29)-en-3-yl acetate	C32H52O2	468
5	17.29	65.7	Lup-20(29)-en-3-yl acetate	C32H52O2	468
6	17.53	70.2	Lup-20(29)-en-3-yl acetate	C32H52O2	468
7	18.04	37.9	1-Heptatriacotanol	C37H76O	536
8	26.39	61.2	Kolavenol	C20H34O	290
9	26.54	99.2	Kolavenol	C20H34O	290
10	27.39	99	α-Amyrin	C30H50O	426
11	28.91	39.8	Cedran-diol (8s,14)	C15H26O2	238
12	30.19	40.5	20-Hydroxy-5α-pregnan-18-oic acid	C21H34O2	334
13	31.16	64.2	α-Amyrin	C30H50O	426
14	33.05	67.1	Lup-20(29)-en-3-yl acetate	C32H52O2	468

### Ligand–receptor interaction

**Figure 6:**
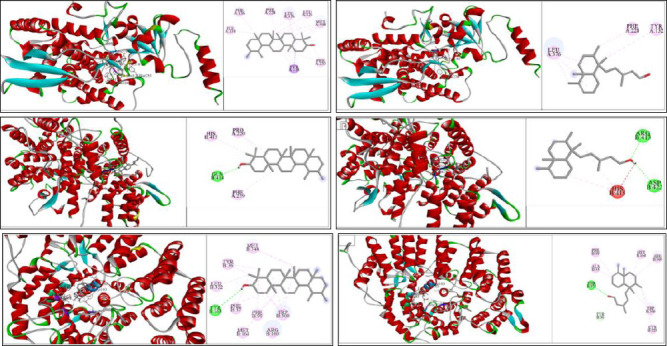
3-D (left) and 2-D (right) structures of proteins of *C. albicans* bound with compounds identified from *A. scholaris* bark. (A) CYP51 with α-amyrin, (B) CYP51 with kolavenol, (C) Farnesyltransferase with α-amyrin, (D) Farnesyltransferase with kolavenol, (E) Geranylgeranyltransferase-I (GGTase-I) with α-amyrin, and (F) GGTase-I with kolavenol.

**Figure 7:**
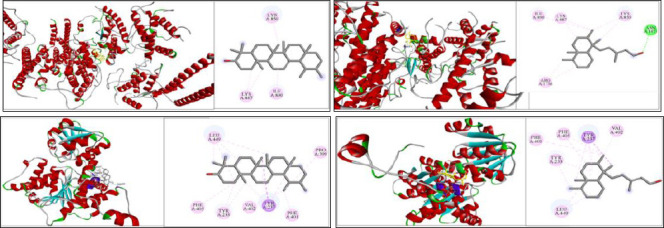
3-D (left) and 2-D (right) structures of proteins of *P. falciparum* bound with compounds identified from *A. scholaris* bark. (A) VAR2CSA with α-amyrin, (B) VAR2CSA with kolavenol, (C) SAHH with α-amyrin, and (D) SAHH with kolavenol.

**Figure 8:**
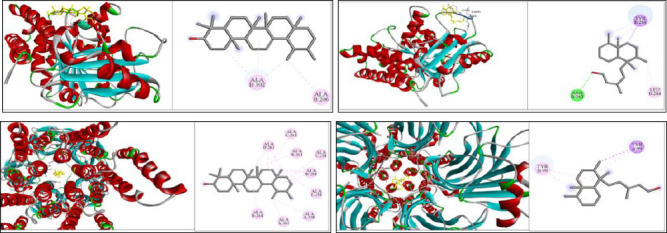
3-D (left) and 2-D (right) structures of proteins of *C. elegans* bound with compounds identified from *A. scholaris* bark. (A) β-Tubulin with α-amyrin, (B) β-Tubulin with kolavenol, (C) Glutamate-gated chloride channel (GluCl) with α-amyrin, and (D) GluCl with kolavenol.

The identified compounds of *A. scholaris* bark extract, α-amyrin and kolavenol, were docked against the vital proteins of pathogenic fungi, malaria, and helminth parasites to gain insight into the probable molecular mechanism and binding efficiency. The protein grid boxes were prepared according to the coordinates specified in Table 5. Exhaustiveness of analysis was set at 8 to dock the ligands to the target proteins of fungi (Table 6), malaria (Table 7), and helminth parasites (Table 8). The molecular structures depicting the ligand–receptor interactions and the exact binding sites are shown in Figures 6–8.

**Table 5:** Molecular data setup for docking in AutoDock Vina, showing grid size, center position, and protein data bank (PDB) accession codes.

Target	Size x	Size y	Size z	Center x	Center y	Center z	PDB code
CYP51	84	56	66	−45.733	−15.004	22.983	5V5Z
Farnesyltransferase	66	80	78	26.559	−36.078	200.098	1V8B
GGTase-I	86	76	70	33.082	41.666	23.232	3DRA
β-Tubulin	64	56	60	−32.679	−7.917	−17.606	7X4N
GluCl	122	94	126	−75.996	−13.595	24.676	4TNV
VAR2CSA	62	68	68	37.859	32.362	56.095	7JGD
SAHH	92	104	126	157.763	159.146	151.655	1V8B

**Table 6:** Molecular binding scores of bioactive compounds identified from *Alstonia scholaris* bark extracts on proteins of *Candida albicans.*

Ligand	CID code	Target	Binding energy (kcal/mol)	Amino acid interaction
α-Amyrin	73170	CYP51	−10.4	Tyr118, Leu121, Iso131, Phe126, Phe228, Phe233, Leu376, Met508
		Farnesyltransferase	−9.1	Pro220, Phe259, Gln414, His415
		GGTase-I	−9.1	Tyr36, Phe37, Phe99, Arg160, Tyr163, Met164, Trp300, Met348, Leu352
Kolavenol	6442554	CYP51	−7.5	Tyr132, Phe228, Leu376
		Farnesyltransferase	−7.1	Arg413, His415, Asp422
		GGTase-I	−9.1	Ala33, Tyr36, Phe99, Arg160, Met164, Cys225, Trp300, Thr375

**Table 7:** Molecular binding scores of bioactive compounds from *Alstonia scholaris* bark extracts on proteins of *Plasmodium falciparum.*

Ligand	CID code	Target	Binding energy (kcal/mol)	Amino acid interaction
α-Amyrin	73170	VAR2CSA	−9.4	Lys850, Lysine887, Ile890
		SAHH	−10.0	Tyr233, Tyr237, Pro399, Phe401, Val402, Phe405, Leu449
Kolavenol	6442554	VAR2CSA	−6.5	Lys850, Lys887, Ile890, Arg1736, Asn1871
		SAHH	−7.4	Tyr233, Tyr237, Phe401, Val402, Phe405, Leu449

**Table 8:** Molecular binding scores of bioactive compounds identified from *Alstonia scholaris* bark extracts on proteins of *Caenorhabditis elegans.*

Ligand	CID code	Target	Binding energy (kcal/mol)	Amino acid interaction
α-Amyrin	73170	β-Tubulin	−8.8	Ala206, Ala302
		GluCl	−9.4	Ala258, Ala261
Kolavenol	6442554	β-Tubulin	−6.6	Tyr281, Arg282, Leu284
		GluCl	−7.1	Tyr99

α-Amyrin was found to have exceptional binding efficiency to the major proteins. It interacts most efficiently with *C. albicans* target proteins. The highest binding efficiency was observed against CYP51 with a binding energy as low as −10.4 kcal/mol and amino acid interactions at seven residues. Its binding energy against farnesyltransferase and GGTase-I was −9.1 kcal/mol each. However, given equal binding energy, GGTase-I showed a higher capacity for molecular interaction, with binding sites at nine amino acid residues, compared with farnesyltransferase, which showed only four amino acid interaction sites. Its binding efficiency was almost equally high against *P. falciparum* VAR2CSA (binding energy −9.4 kcal/mol) and SAHH (binding energy −10 kcal/mol), but with fewer amino acid interactions. It also indicated high-affinity binding to *C. elegans* GluCl, with a binding energy of −9.4 kcal/mol, but comparatively lower binding to β-tubulin, with a binding energy of −9.4 kcal/mol.

Kolavenol was less efficient than α-amyrin, showing moderate binding across all proteins except GGTase-I, against which it was highly efficient (binding energy −9.1 kcal/mol).

## DISCUSSION

### Broad-spectrum anti-infective relevance of *A. scholaris*

We found that *A. scholaris* bark contains bioactive compounds that exhibit cross-ranging activities against pathogenic fungi, malarial, and helminth parasites. While many anti-infective medications have only specific activity against selected pathogens, the most important antimalarials are demonstrably broad-spectrum. Quinine and its derivatives, quinine, quinidine, and quinacrine, significantly reduced the worm burden and egg-laying of the blood helminth *Schistosoma mansoni*, the most prevalent helminth parasite of humans [34]. Several quinoline derivatives were shown to inhibit the motility and development of *Haemonchus contortus*, an intestinal nematode of ruminants [35]. There are reports that quinoline-containing plants, such as *Acmella* and *Zanthoxylum* species, effectively kill various helminth parasites [36]. Chemically modified quinoline compounds exhibit effective antimicrobial activity against important pathogens, including the bacterium that causes tuberculosis [37], as well as cancer cells [37].

### Pharmacological parallels with antimalarial and anthelmintic drugs

The currently used antimalarials, artemisinin and its related compounds, have been extensively shown to have a wide range of anthelmintic activity, including efficacy against a host of helminth parasites such as nematodes, trematodes, and cestodes [39]. They are not only shown in clinical trials to be useful against all forms of schistosomiasis, but they are also effective against carcinogenic helminths, *Clonorchis sinensis* and *Opisthorchis viverrini* [40], as well as several pathogenic fungi and bacteria [41, 42]. On the other hand, standard anthelmintics such as albendazole and mebendazole are known to have anticancer and antiprotozoal activities [43]. Recent studies have shown that albendazole and its derivatives are effective against various life-cycle stages of *P. falciparum* [44].

### Antifungal, antimalarial, and anthelmintic implications

Our findings indicate that the antimicrobial, antimalarial, and anthelmintic activities of *A. scholaris* bark extract can be understood from the pharmacological properties of these antimalarial and anthelmintic drugs. The plant extract showed good activity against the cestode and fungi tested. It is notable for its efficacy against pathogenic fungi designated as “fungal priority pathogens” [45], which are largely underexplored in medicinal plants. In particular, the most susceptible species, *N. keratoplastica*, causes a range of diseases, from skin infections to organ failure, in different animals [46]. It was consistently as efficacious as albendazole (p > 0.05) at all concentrations tested against the cestode. Anthelmintic resistance is most prevalent in veterinary animals [47]; hence, the plant offers a promising solution to the major economic burden in animal farming. However, its antimalarial activity was of moderate efficacy (IC_50_ between 10–50 µg/mL). This could be due to geographical variations and the mode of extraction of the plant species, as variable antimalarial efficacy has been reported from different parts of the world. Ethanolic extract of the specimen from West Timor, Indonesia, showed an IC_50_ of 15.6 µg/mL against 3D7, with lupenyl acetate as its major compound [48], whereas the specimen from Sumatra showed weak activity (IC_5_0 > 50 µg/mL) against 3D7 [49]. The methanolic extract of the Thai specimen exhibited negligible activity, with an IC_50_ of 181.4 µg/mL against K1 [50]. The major bioactive compounds in different *Alstonia* species are indole alkaloids, with echitamine among the best-studied. These alkaloids exhibited only moderate antimalarial efficacy, with IC_50_ values ranging from 11 to 45 µg/mL against various *P. falciparum strains* [51, 52]. Thus, the broad-ranging anti-infective activity of *A. scholaris* variety is a novel finding and distinct from studies from other parts of India and Asia. Furthermore, a low resistance index of 1.05 is a novel finding for *A. scholaris* extracts against multidrug-resistant *P. falciparum*, underscoring the potential to overcome antimalarial resistance.

### Role of triterpenoids and chemotype-specific activity

We identified kolavenol and α-amyrin as the major compounds of *A. scholaris*. Kolavenol is a diterpenoid known to be an active principle isolated from plants such as *Eupatorium adenophora*, *Entada abyssinica*, and *Kaempferia* species. It is highly potent as an antiprotozoal compound against *Trypanosoma brucei rhodesiense*, the deadliest form of trypanosomiasis [53]. It has been shown to have anticancer effects on different carcinomas in mice [54], and to be highly cytotoxic against cancer cell lines while also exhibiting antibacterial activity [55]. α-Amyrin is a pentacyclic triterpenoid identified in several plants. It is widely recognized for its strong antihyperglycemic effect [56, 57] and anti-inflammatory action [58]. Other effects, including analgesic, antidepressant, gastroprotective, immunomodulatory, and hypolipidemic activities, are also documented [59, 60]. The predominance of α-amyrin and kolavenol, rather than indole alkaloids in other chemotypes, may explain the moderate but consistent multi-target activity and non-toxicity of the Mizoram variety, suggesting adaptation to the Indo-Burma hotspot environment.

### Computational support and future directions

Although it is not conclusive that the compounds identified are directly responsible for the multifaceted pharmacological activities of *A. scholaris*, the computational data support the general effects. Particularly, the high binding affinity of α-amyrin to key enzymes across kingdoms, such as −10.4 kcal/mol on fungal CYP51, −10.0 kcal/mol on malarial SAHH, and −9.4 kcal/mol on helminth GluCl, provides the first computational rationale for triterpenoid-driven broad-spectrum effects in the Mizoram variety of *A. scholaris*. Further molecular modeling, fractionation with isolation and individual-compound testing, including *in vivo* assays, will be essential for understanding precise binding interactions and other pharmacological properties. Our findings thus provide evidence that the Mizoram variety of *A. scholaris* is a source of lead molecules for the development of efficacious pharmaceutical drugs against a broad range of pathogens.

## CONCLUSION

The present study demonstrates that *A. scholaris* bark extract possesses broad-spectrum anti-infective potential, exhibiting antifungal, antimalarial, and anthelmintic activities. The extract showed significant inhibitory effects against *N. keratoplastica* and *C. albicans*, while comparatively lower efficacy was observed against *A. fumigatus*. In the antimalarial assay, the extract exhibited moderate activity against both drug-sensitive (3D7) and multidrug-resistant (K1) strains of *P. falciparum*, with a notably low resistance index (Ri = 1.05), indicating minimal cross-resistance. Furthermore, the extract demonstrated considerable anthelmintic efficacy against *R. echinobothrida*, with effects comparable to albendazole (p > 0.05). Importantly, cytotoxicity analysis confirmed the safety of the extract, with CC_50_ values > 100 µg/mL against Vero cells. Phytochemical profiling revealed the predominance of triterpenoids, particularly α-amyrin and kolavenol, which were further supported by molecular docking studies showing strong binding affinities with key target proteins across fungal, protozoan, and helminth systems.

From a practical perspective, these findings highlight the potential of *A. scholaris* bark as a promising source of multi-target therapeutic agents, particularly in the context of increasing antimicrobial and anthelmintic resistance. Its comparable efficacy to standard drugs and low cytotoxicity suggest suitability for further development in veterinary and possibly human medicine, especially for integrated management of parasitic and fungal infections in resource-limited settings.

A major strength of this study lies in its comprehensive approach, integrating *in vitro* biological assays with chemical characterization and computational modeling, thereby providing a multi-level understanding of the pharmacological potential of the extract. Additionally, the inclusion of multidrug-resistant *P. falciparum* and priority fungal pathogens enhances the translational relevance of the findings.

However, several limitations must be acknowledged. The study was restricted to evaluating crude extracts without isolating individual active constituents, which limits the precise attribution of bioactivity. The moderate antimalarial efficacy indicates that optimization of extraction methods or compound enrichment may be required. Furthermore, the absence of *in vivo* validation and pharmacokinetic assessment restricts direct clinical applicability. Variability due to geographical origin and chemotypic differences of *A. scholaris* also warrants further investigation.

In conclusion, *A. scholaris* bark extract represents a promising candidate for the development of novel broad-spectrum anti-infective agents. The combined evidence from biological assays and molecular docking supports its multi-target pharmacological potential. Future studies focusing on compound isolation, mechanistic validation, and *in vivo* efficacy will be essential to translate these findings into practical therapeutic applications.

## DATA AVAILABILITY

The data used to support the findings of this study are included within the manuscript.

## AUTHORS’ CONTRIBUTIONS

LNM: Sample collection, extraction, antifungal assay. PB and LNM: Anthelmintic assay, GC-MS and first draft of the manuscript. BL and PB: Molecular docking and statistical analysis. KL and PB: Antimalarial assay. LN: Project conception, resource acquisition and supervision. KL: Fund acquisition, conceptualization, data interpretation and manuscript finalization. All authors have reviewed the manuscript and approved it for publication.
